# The Cybernetics of Mental Disorders as a Bridge Between Palliative Care and Palliative Psychiatry—A Narrative Review

**DOI:** 10.3390/healthcare14142053

**Published:** 2026-07-08

**Authors:** Michael Brinkers, Beatrice Thielmann, Irina Böckelmann

**Affiliations:** 1 Pain Outpatient Clinic of the Department of Anesthesiology and Intensive Care, Faculty of Medicine, Otto von Guericke University Magdeburg, Leipziger Str. 44, 39120 Magdeburg, Germany; michael.brinkers@med.ovgu.de; 2 Institute of Occupational Medicine, Faculty of Medicine, Otto von Guericke University Magdeburg, Leipziger Str. 44, 39120 Magdeburg, Germany; irina.boeckelmann@med.ovgu.de

**Keywords:** decay, depression, identity, nonlinearity, quality of life, schizophrenia, stability, systems theory

## Abstract

**Background:** Palliative psychiatry as a new subfield of practice focuses on the care of patients with severe, chronic mental diseases. The aim is to improve the quality of life of those affected and alleviate their suffering. The cybernetic approach can be used to achieve these goals. **Materials and methods:** This narrative study aims to present palliative psychiatry from a cybernetic perspective. To this end, it examines how and whether these concepts are already being used in related fields such as palliative care and general psychiatry. Finally, it discusses the implications of this for palliative psychiatry. The focus is also on schizophrenia and depression. Cybernetic terms were entered along with the terms “schizophrenia” and “depression” in the PubMed and PubPsych databases. In addition, articles and books on psychological terms, such as affect logic, vulnerability, and comorbidity, were used in a snowball system. **Results:** In recent decades, general psychiatry has dealt with all of the cybernetic terms examined in this study. Palliative psychiatry has only been oriented towards palliative care and has used its cybernetic terms. However, no other cybernetic terms were used. Articles on palliative care for patients with schizophrenia show that palliative psychiatry initially addresses problems that have been known for over ten years. In the case of depression, only studies on palliative care outside the field of psychiatry have been published. **Conclusions:** The integration of cybernetic concepts could provide palliative psychiatry with a theoretical foundation that goes beyond the previous borrowings from palliative care. This opens up new possibilities for better understanding complex disease progression, especially in schizophrenia and depression, and providing therapeutic support.

## 1. Introduction

A new field of medicine has recently emerged: palliative psychiatry. This concept is concerned with caring for patients with severe mental disorders (severe and persistent mental illness, or SPMI). According to the National Institute of Mental Health (NIMH), SPMI is defined as a mental health diagnosis that meets three criteria [1]:

- Diagnosis: A major affective nonorganic psychotic disorder or a disorder that may lead to a chronic disability.
- Disability: A severe, recurrent disability of basic living skills and participation due to mental disorders (five criteria).
- Duration: (a) The person has undergone psychiatric treatment more than once in their life in a more intensive manner than outpatient treatment; (b) the person has experienced an episode of continuous, supportive residential care that was not a hospital stay but lasted long enough to significantly disrupt their normal life situation [2].

This also includes the definition of palliative care, which addresses the treatment of patients with incurable, advanced disease and aims to maintain or improve the quality of life of affected patients [3]. According to Trachsel and coauthors, this includes patients with severe chronic schizophrenia and inadequate quality of life, with treatment-resistant depression and repeated suicide attempts, and with severe, long-standing treatment-resistant anorexia nervosa [3]. It includes patients with other affective disorders, paranoia, organic or psychotic disorders, personality disorders, or other mental disorders that can lead to chronic disability [2]. Addictions to addictive substances are also included [4].

Initially, Lindblad et al. [5] and Trachsel et al. [3] attempted to transfer palliative care practices to palliative psychiatry. However, they refer exclusively to psychological symptoms, even though depression, for example, can also cause physical symptoms such as pain [6,7]. According to Cornwall et al. [7] and Paykel et al. [8], in most cases, physical symptoms are the main reason for readmission. Various authors have discussed physical symptoms for years [9,10]. This approach considers both the physical and psychological symptoms associated with cybernetic items.

This article does not draw a direct comparison between palliative care and the concept of PP. Instead, a relationship between palliative care and general psychiatry is established through a common third concept, cybernetics, to describe the new subfield of “palliative psychiatry.”

To understand the cybernetic approach used to describe palliative psychiatry, explanations of the meaning of cybernetics are necessary. A study on cybernetic principles and their significance for palliative care [11] described the cybernetic roots of palliative care terms. These include quality of life, the cause-and-effect model, the biopsychosocial model, symptom control, and autonomy.

In addition, terms have been established that are cybernetic but are not or are rarely part of the palliative care discussion [11,12]. These include complexity, stability/identity, time (decay/temporalization, preformation/anticipation, and rhythm), nonlinearity, and fuzziness. These terms are now used for research in psychiatry, a field that is older than palliative care.

The following distinctions are made:

e.g., (1) Terms previously used in palliative care (quality of life, cause-and-effect model, biopsychosocial model, symptom control, or autonomy) are listed.

(2) Terms not previously used in the palliative care literature (complexity, stability/identity, time (decay/temporalization, preformation/anticipation, rhythm), nonlinearity, and fuzziness) are listed.

(3) Other terms, such as control loop, dualism, self-regulation, autopoiesis, controller, resources, feedback control, and anticipation.

This review aims to compare the cybernetic terms described in palliative care with those used in the general psychiatric literature (on the basis of the psychiatric disorders of schizophrenia and depression). In this way, statements can be made about the resulting consequences of cybernetic principles in palliative psychiatry, and possible conclusions for palliative psychiatry can be made.

## 2. Materials and Methods

The manuscript is a narrative review of cybernetic concepts in palliative psychiatry.

**Figure 1:**
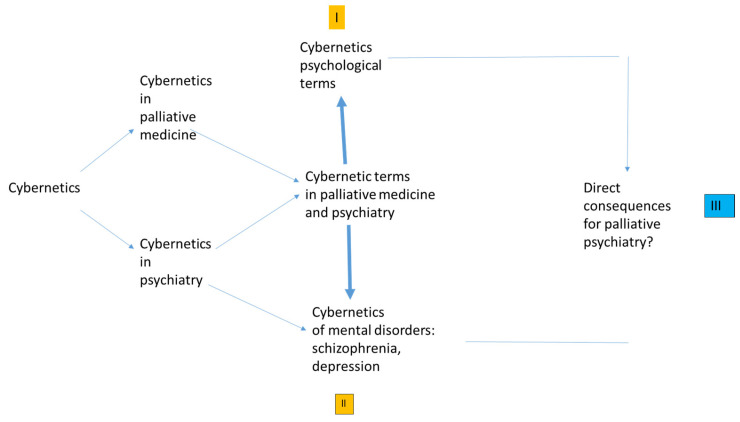
Illustration of the procedure used in the review of cybernetic concepts up to palliative psychiatry (Parts I–III).

A literature review and a three-part analysis were conducted with corresponding questions to compare the cybernetic terms used in palliative care and general psychiatry (based on the literature of cybernetic terms in schizophrenia and depression). Figure 1 illustrates the methodology employed for this narrative review. Initially, articles addressing cybernetic concepts in palliative care and general psychiatry were included. From these, we filtered out, on the one hand, the cybernetic concepts underlying terms such as ‘symptom management’, ‘identity/autonomy’ and ‘structure’ (I), and, on the other hand, the cybernetic concepts appearing in the context of the clinical pictures of schizophrenia and depression (II).

Finally, we drew conclusions about the implications of using cybernetic concepts to characterize palliative psychiatry (III).

I. Inclusion criteria: The basis was the cybernetic terms previously examined for palliative medicine and established by Brinkers and coauthors [11] (quality of life, cause-and-effect model, biopsychosocial model, symptom control or autonomy, complexity, stability/identity, time (decay/temporalization, preformation/anticipation, rhythm), nonlinearity and fuzziness) in concepts of general psychiatry.

II. This also includes cybernetic concepts and regulatory functions (concepts such as a control loop, dualism, self-regulation, autopoiesis, regulator, resources, feedback control, anticipation) in publications on mental disorders in general and their significance for schizophrenia and depression in particular.

III. The aim of this thesis is to answer the following question: What are the consequences of applying cybernetic concepts to understanding, modeling, and therapeutic practice?

A literature search was conducted on all of the above terms in connection with “schizophrenia” or “depression” in the PubMed and PubPsych databases from 1990 to 2025, including German and English-language publications. PubPsych included references from all areas of psychology, including those from Europe. PubPsych indexes databases such as PSYNDEX, PsychOpen, PsychData, ISOC-Psicologia, MEDLINE, PASCAL, NARCIS, NORART, ERIC, and others.

The terms “cybernetics” and “cybernetic principles” were combined with the terms “psychiatry,” “schizophrenia,” and “depression.”

The cybernetic terms were divided into three groups:

(a) Cybernetic terms frequently used in palliative medicine, such as “biopsychosocial,” “quality of life,” “symptom”/“symptom treatment,” “cause and effect,” and “autonomy.”

(b) Terms that are rarely used in palliative medicine or have no equivalent, such as “nonlinearity”, “complexity”/“interconnection”, “structure”, “identity”/“stability”, and “time” (“decay”/“temporalisation”, “preformation”/“anticipation”, and “rhythm”).

(c) Items from the control loop, such as “regulator,” “controller,” “feedback control,” “self-regulation,” “dualism,” “autopoiesis,” and “anticipation.”

The noncybernetic terms “palliative care” and “genetics” were included. Exclusion criteria: There were no exclusion criteria for articles. The aim was to determine whether cybernetic items were described in the fields of palliative care and general psychiatry. 

The starting point was the small group of key specialist (such as the groups of authors led by Trachsel, Lindblad or Westermair) articles. In addition, reference was made to textbooks on psychiatry and online resources on the subject. The snowball method was used to identify relevant literature (since 1970) on psychological terms such as “affect logic,” “vulnerability,” or “comorbidity” to determine what has been written about cybernetic terms under these headings. The terms “residue,” “therapy resistance,” and “revolving door patients” were also included. Thus, the body of literature on palliative psychiatry, along with “schizophrenia” and “depression”, was gradually expanded.

## 3. Results

### 3.1. General

The results are based on the specialist literature (full text) identified and evaluated in the literature search.

A systematic search of the PubMed and PubPsych databases yielded many relevant publications on the subject (search date 18 February 2026). Table 1 and Table 2 present an overview of the key results.

A search of the PubPsych database yielded the following results:

As the two tables show, individual cybernetic terms frequently appear in publications. However, it is also important to consider how often these terms are used in the context of palliative care and general psychiatry. Owing to the large volume, an initial preselection was made. Of this preselection, those deemed sufficiently explanatory (i.e., publication date in the last decade or scope of discussion) were downloaded. In addition, the snowball method was used to include sources (cross-references from articles and older sources from authors already used). This resulted in 140 literature sources (138 articles, one master’s thesis, and one care report).

The total number of literature sources ultimately used for the evaluation was 56. These were supplemented by two dissertations or diploma theses, three lectures and 27 books on psychiatry and individual topics such as affective logic or nonlinearity.

### 3.2. Results in Detail

General principles: Brinkers et al. described four critical points of cybernetic systems in their publication on cybernetics in palliative care [11]:

1. Cybernetic systems strive to counteract disturbances. However, in slow and long-lasting process statements, they also make predictions. These can be incorrect.
2. Cybernetic systems are finite (decay processes). Errors can occur during restructuring.
3. Cybernetic systems work drastically slowly. If disturbances are counteracted as a result, these systems die.
4. Cybernetic systems are in a constant process exchange/adaptation with an environment that is different from them. The system dies if the conditions in the environment are identical to those of the system or if there is no information content in the environment at all (excessively low stimulus).

#### 3.2.1. From Cybernetic Terms in Palliative Medicine to General Psychiatry (Part I)

Brinkers et al. examined which types of palliative care follow cybernetic principles and which do not [11]. The authors considered biopsychosocial factors, quality of life, symptoms, and their treatment, as well as autonomy and cause-and-effect relationships. Nonlinearity, complexity, and interconnectedness; questions of structure, identity, and stability; and the factor of time were also included in the analysis.

As Table 3 shows, all cybernetic items have already been adopted by general psychiatry but not by palliative care. Table 3 summarizes and compares the key findings of the literature review. Particular attention is given to the points discussed by Brinkers and coauthors in palliative care [11].

The results presented in Table 3 show the characteristics of cybernetic systems. The human system is operationally closed, has its own procedures, and is therefore autonomous.

However, as an individual ability, “autonomy” can be effective only if it is integrated into a relational context. Therefore, the system is simultaneously connected to the environment (“biopsychosocial”). Its individual subsystems are interconnected (“complexity”). Their actions and the connections between them work in a “nonlinear” way, which is why there is no one-to-one relationship between “cause” (external disturbances) and “effect” (symptoms). Therefore, the goal of therapy is not to eliminate symptoms but to achieve a new balance based on QoL.

#### 3.2.2. Cybernetic Terms in the Literature on Schizophrenia and Depression (Part II)

Following the presentation of the results from publications on mental disorders in general, we now turn to publications on cybernetic concepts in schizophrenia and depression in particular. These cybernetic concepts are also discussed in the context of PM [11].

##### (A) Control Terms Loop

These terms are the basics of cybernetics: control loops, dualism, self-regulation, autopoiesis, counterregulation/resources, and anticipation.

**Role of the regulator/control loop**

**Figure 2:**
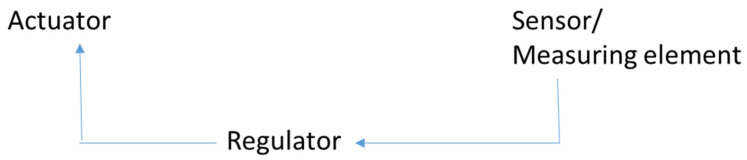
The representation of the control loop.

Psychiatry has long been concerned with cybernetics and systems theory. Vossius described the cybernetic system of the control loop. This consists of a variable that is measured and altered by a disturbance factor. The sensor detects this change and transmits it to the controller, which sends a signal to other parts of the system to make the necessary adjustment. In the case of blood pressure, for example, this would involve adjusting vascular resistance. The result of this change is then relayed back to the sensor. This creates a feedback loop which, ideally, cancels out the disturbance factor. Feer described depression and schizophrenia as the result of a disturbance in the control loop in a book on cybernetics in psychiatry [39]. Figure 2 shows a simplified representation of the control loop.

A system is not necessarily limited to a physical organ. Humans themselves can also be viewed as systems.

**Afferent to the controller in patients with schizophrenia**

*Anticipation and feedback control:* According to Feer, feedback control by the actuators commissioned by the controller is usually insufficient for mental processes [39]. Rather, the system needs the ability to anticipate that it is prepared for a disturbance before it even occurs. Anticipation is geared towards long-term processes [39]. However, life-threatening disturbances require faster feedback control.

*Anticipation of danger:* This applies, e.g., to the moment of a possible, suspected, but unknown danger. The organism is alerted. It avoids any assumption about the direction from which danger might arise. Only then is it possible to react to any danger at maximum speed. If the situation lasts only a short time, the individual’s reaction returns to normal. However, if it lasts longer than a few minutes without any external cause, the reaction, which is characterized by drowsiness and heightened sensitivity to sensory stimuli, is similar to the onset of acute paranoid schizophrenia.

*Inappropriateness of anticipation:* Another regulator influences, according to Feer, the regulator itself [39]. According to Vossius, the regulator derives its current setpoint and sensitivity from this model [40]. This also applies to anticipation, i.e., the prediction of events in mental disorders: whether a window in the neighboring house is open or closed is irrelevant to a healthy person and therefore insignificant; the person is not particularly surprised. However, if an individual is fixated on observing his or her surroundings for pathological reasons, an open or closed window is no longer a matter of course but an event with a certain probability. A person who abnormally assesses the probability of numerous everyday events as low experiences these events as a surprise when they occur. This is inversely proportional to probability.

If the window in the neighboring house is now open and the residents have left, according to Feer [39], this means the following:

This, for the fixed observer, is an unexpected event, as the departure was not agreed on with him. Culturally agreed-on signals are called signs (e.g., traffic lights, indicators, and calls for help). This window was not agreed on. This is called a note/indication [39]. What does it signify, however, in this specific case? Notes require interpretation. A healthy person interprets a note (i.e., an objectively unexpected event), such as a bang, as a sign of an accident or a supersonic aircraft. On the other hand, a mentally ill person experiences even an open window as unexpected: Are there thieves in the house? Have the residents returned unexpectedly? Was the window not secured properly and did the wind blow it open? In addition to the misinterpretation that an everyday event is unexpected, people with mental illness note that they experience an overwhelming number of unexpected events. Therefore, on the one hand, they must make a selection; on the other hand, they must reinterpret the overall situation. The interpretation of the situation affects the interpretation and vice versa [39]. This creates a delusional mood.

According to Feer, the disturbed probability also applies to thoughts, associations, and ideas: “Above all, the unwanted, undesirable and unwelcome ideas are so improbable that they severely interrupt the continuous train of thought. As a result, they are experienced as foreign and imposed from outside.“ [39]. This can be experienced as the influence of others’ will or the sense that one’s own ideas/thoughts are no longer perceived as one’s own and are no longer perceived as thoughts at all; they are something new in the sense of hallucinations [16].

**The influence of the regulator in depression**

The regulator also provides the necessary motivation, drive, or energy for movement. If muscle movements (anatomical efferents) are part of behavior, then the efferent part of the control loop (i.e., the regulator and actuator) is also important. As is usually the case in cybernetic systems, this involves energy management.

No organism can remain active indefinitely. When fatigued, it must rest. Hell [2005] refers to a defensive adaptation process in situations of unmanageable stress [41], and sees this as reducing a hopeless struggle, a purposeless flight or disintegration. Biologically sensible rest must inhibit an organism’s activity in a differentiated manner, i.e., it must occur in cases of fatigue but not in dangerous situations. This is achieved through motivation, either gain or loss.

If the regulator or its upstream structures become diseased, the actuator is no longer controlled and assumes an extreme value—either extremely large or extremely small. This means high energy loss or very low energy consumption. However, according to Feer (1970), this also means high or very low motivation or incentive to be active [39]. Depression leads to low motivation. This is accompanied by the feeling that losses become more significant and gains are not adequately considered. One works because one must. One cannot hope for a gain. If a gain does occur, it is insipid.

Depression, as a pathologically heightened protective posture, first manifests as will inhibition. According to Hell, people with severe depression are primarily affected by changes in their thinking and volition [41]. The resulting reduction in psychological energy reduces the loss of physical energy, resulting in a positive energy balance. However, reduced motivation leads to a negative attitude towards positive life events when negative experiences are exaggerated. This can lead to both depressive moods and delusions. In contrast to schizophrenic delusions, these are not about the interpretation of improbable events but about the pathologic certainty that everything unfavorable and harmful is more probable and significant than anything favorable.

The cybernetic view of malfunctions in the control loop partially dispels the notion that these are faults in a physical apparatus [41].

***Dualism***

Cybernetic processes/control loops are considered energetically open (information) but operationally closed (biochemical, genetic, and psychodynamic processes) [11]. According to Maturana, operational closure requires a suitable environment [42].

***Autopoiesis***

According to Maturana (1985), autopoiesis is the mechanism that makes living entities autonomous [42]. Autopoiesis is one aspect of the diverse research approaches to schizophrenia. This raises the question of how deviance processes (the reality of schizophrenic patients compared to that of the environment) can be explained by the subjective appropriation or construction of reality [43]. Here, we refer primarily to Maturana, who described the concept of autopoiesis with cognitive self-organization. With regard to depression and schizophrenia, autopoiesis means that the mental disorder was generated within the subject and not externally. Self-organization goes hand in hand with constant interaction with the environment. Therefore, the question is how and by what means the psyche does this.

According to Conrad [44] (cited in [43]), a process of “displacement” then occurs in patients with schizophrenia. Among other things, there is a departure from the common sign system. The resulting lack of external information is then replaced by internally guaranteed security [43,45].

***Counterregulation and resources***

Older research has shown that in depression, overactivity of the limbic system is no longer sufficiently counterregulated by the cortical areas, or the connection between the two areas is broken. This leads to underactivity of the frontal lobe (prefrontal cortex) [41].

##### (B) Cybernetic Items Frequently and Rarely Used

In relation to the second question (cybernetics and the literature on depression and schizophrenia), the literature on general psychiatry in relation to these two disorders provides descriptions that no longer play a role in palliative medicine. Table 4 provides an overview of the most important findings from the literature review on cybernetics, depression, and schizophrenia. At the beginning of Table 3, we discussed the cybernetic terms used in psychiatry and palliative care. Simultaneously, we examined the specific literature on schizophrenia and depression (Table 4).

In summary, the results presented in Table 4 show that publications on cybernetics in schizophrenia (and, to some extent, also in depression) demonstrate how closely cybernetic terms are related even more strongly than in general psychiatry; e.g., concepts such as “fluctuations” or “disintegration of the mental structure/system” in patients with schizophrenia are related to “identity” and mental “stability” disturbance.

Autopoiesis goes hand-in-hand with constant interaction with the environment (“biopsychosocial”). “Autopoiesis” means that schizophrenia and depression are generated by the individual. The disturbed “regulator” plays an important role in this process. This is not primarily a matter of a single disturbance process but rather a failure of “counterregulation,” i.e., the mechanisms that control each other. This has also been demonstrated by recent genetic research [46]. Owing to the Ptolemaic shift in self-centeredness [44], “autonomy” and “networking” are turning into their opposites: energy openness decreases, and operational closedness increases. However, the underlying “vulnerability” of individuals, which varies in degree, leads to unpredictable results when an additional external disruptive factor (“nonlinearity”) comes into play.

Narrowing the scope of general psychiatry down to the clinical manifestations of schizophrenia and depression makes it clear that all cybernetic concepts relating to mental disorders have already been discussed.

#### 3.2.3. Consequences of Cybernetics for Palliative Psychiatry (Part III)

Table 3 and Table 4 show that general psychiatry has dealt with (almost) all cybernetics concepts. Palliative care focuses only on the concepts of the “biopsychosocial”, “quality of life”, “symptoms/symptom control”, “cause and effect” and “autonomy”. Palliative care rarely, if ever, addresses other cybernetic concepts, such as “non-linearity”, “complexity”, “structure”, “stability”, “identity” or “time”.

**Figure 3:**
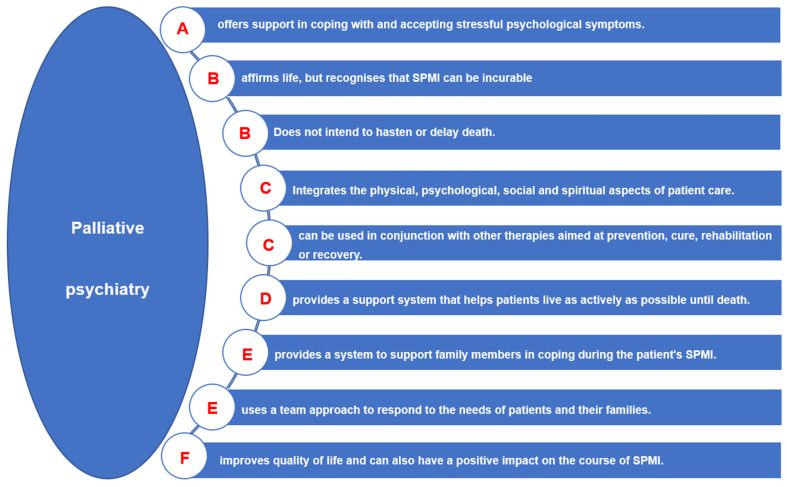
Presentation of the characteristics of palliative psychiatry according to Lindblad et al. 2019 [4] and assignment to the 6 points of palliative psychiatry (A—symptoms, B—curability, C—networking/complexity, D—autonomy, E—autonomy, biopsychosocial, F—quality of life).

In contrast, the characteristics of palliative psychiatry listed by Lindblad et al. [2019] should be considered [5] (Figure 3).

The characteristics of palliative psychiatry presented by Trachsel et al. [3] and by Lindblad et al. [5] can be [5] classified under six headings (Figure 3):

(A) Symptoms
(B) Curability and symptom control
(C) Networking/complexity
(D) Autonomy
(E) Autonomy and biopsychosocial
(F) Quality of life

These are addressed in the Section 4.

**Table 4:** List of important statements from the literature review for cybernetic terms, differentiated according to cybernetics and general psychiatry (schizophrenia and depression).

Cybernetic Terms	Schizophrenia	Depression
**Cybernetics and literature on depression and schizophrenia**		
**Biopsychosocial**	No single factor leads to depression or schizophrenia. When a disruptive factor occurs, the important thing is the condition of the individual patient [27] and his or her ability to counteract it. This is the product of protective factors or resources and stress factors [47].	
**Quality of life (QoL)**	In a survey of 565 schizophrenic patients and 605 psychiatrists, the psychiatrists described the quality of life of patients with schizophrenia as the absence of disabilities and impairments due to the illness and the importance of appropriate professional help and self-help [48]. However, the patients associated psychiatric treatment with quality of life only in exceptional cases and then only “not having to take medication” [49].	
**Symptoms**	Somatic symptoms are part of the diagnosis of schizophrenia and depression.	
		Since 2002, authors have repeatedly pointed to additional physical symptoms [9,10]. Three main areas emerge [50]: 1. Pain 2. Difficulty breathing 3. Palpitations/racing heart.
**Symptom control**	Depression and schizophrenia are not treated to the maximum extent so that patients no longer feel anything. Rather, even in cases of depression, periods of depressive moods may occur. However, the patient should be able to easily recover from this mood [14].	
**Autonomy**	Autonomy in schizophrenia (operative unity) is reversed in a negative direction. The subject’s perception revolves solely around themselves or themselves. Everything exists only because of the individual. Conrad refers to this as a “Ptolemaic shift” [44]. Similar to Kratky [22], Folk [51] states, “It must be respected that even ‘wrong’ decisions made by patients that do not correspond to the moral concepts of the nursing staff must be accepted.”	In the case of depression, autonomy can also turn negative in the context of suicidal tendencies. According to Ringel, suicidal tendencies begin with narrowing (tunnel vision): 1. Situational narrowing (personal possibilities); 2. Dynamic narrowing (resignation and late depression); 3. Restriction of Interpersonal Relationships; 4. Restriction of values [52].
**Cause and effect**	Schizophrenia development (and depression) has multiple causes. Individual predisposition (known as the diathesis-stress model [27]), which can also be genetic in nature [46,53], as well as the ability to counterregulate or deal with stress individually [47], is the decisive factor.	
**Nonlinearity**	The basic assumption of vulnerability is that a disruptive factor does not automatically lead to the development of schizophrenia, nor does it affect all people to the same extent [27,47].	
**Complexity of systems/** **Interconnection**	In schizophrenia and depression, the combination of vulnerability, medical history, and acute stress factors that leads to the development of the disorder [27].	
**Structure**	The concept of structure is understood differently in schizophrenia and depression.	
	Current research on schizophrenia examines the structure of the brain [54].	In depression, the term “structure” initially refers to the daily structure (see also “Rhythm” below) [14].
**Identity**	Schizophrenia in particular causes identity disorders, which manifest in (a) ego disorders and (b) delusions. (a) Due to disturbances in the activity, unity, continuity, and independence of the ego [34], patients report thought insertion, thought withdrawal, thought broadcasting, and influence of will; (b) These are concretizations of the changes experienced by the individual during psychosis. In delusions, delusional thinking is linked to biographic memories and cultural symbols [14].	Symptoms of identity disorder, such as derealization and depersonalization, are also found in depression.
**Stability**	Identity disorders, such as those that occur in schizophrenia and depression, are closely linked to the identity of both types of disorder [34].	
	Promoting compliance in patients with schizophrenia is an important contribution to the stabilization of the disease and forms the basis for long-term medication. With long-term medication, significantly fewer patients suffer a relapse than patients with discontinued therapy [55].	One method for maintaining the stability of patients with depression is to stabilize the success achieved through relapse prevention [56].
*The last group of terms is mainly dealt with directly in publications on depression and schizophrenia but not on PM.*		
**Ti** **me factor**	A change in the objective perception of time is most commonly observed in patients with schizophrenia [36].	Patients with depression have a slowed perception of time and altered perception of space [41,57,58].
**Decay**	According to Danzinger (2020), physical symptoms of decay include dismemberment, poisoning, lifelessness, external control, and thin-skinnedness [59].	A depressive reaction no longer appears possible when emotional and cognitive processing is severely impaired due to structural damage to the brain [41].
**Rhythm**	As part of the disruption of circadian rhythms, schizophrenia also causes sleep disorders [60].	Diurnal and seasonal fluctuations are possible in depression [14,41].
**Oscillation**	Patients with schizophrenia exhibited pathologic changes in neuronal oscillations in the theta frequency band during the resolution of cognitive conflicts (disturbance of control function) (Klaiber et al.).	No similar evidence has been found for depression [61].
**Fluctuation**	Frequently, Diefenbacher et al. (1993) noted that chronic and remitting courses of schizophrenic psychosis may differ in that remitting courses are associated with a marked decrease in neurological “soft signs” (subtle deviations from the neurological norm that have been observed in schizophrenia, among other conditions) [62].	
**Anticipation**	Mental anticipation is disadvantageous in the case of anxiety (including in the context of schizophrenia and depression [38]).	

## 4. Discussion

This study addresses the extent to which cybernetic principles can serve as a suitable theoretical foundation for the nascent discipline of palliative psychiatry. While palliative care has drawn on a broad repertoire of concepts and methods aimed at improving quality of life for people with incurable diseases for decades, palliative psychiatry is still in its infancy. Here, the cybernetic perspective enters. It makes it possible to integrate the concepts established in palliative medicine into a broader systems theory framework and, at the same time, incorporate concepts that have been considered only marginally in palliative psychiatry, such as nonlinearity, complexity, stability/identity, or temporality. This approach offers the opportunity to understand disease processes not as linear-causal but as dynamic and self-regulating, particularly in the analysis of severe and persistent mental illnesses such as schizophrenia and depression and disturbance-prone systems.

### 4.1. Critical Examination of Six Points of Palliative Psychiatry

Six points of palliative psychiatry (Figure 3) are critically discussed below from a cybernetic-psychiatric perspective.

#### 4.1.1. Regarding (A) Symptoms

This area has two aspects: selection and regulation of symptoms. In their article (see also the table in Lindblad et al. [5]), Trachsel et al. list only the psychological symptoms of severe mental disorders [3]. However, the problem with this is that psychiatry only considers the psychological symptoms of depression and schizophrenia. This not only overlooks the fact that patients keep coming back because of disturbing, untreated somatic symptoms (such as pain) but, namely, also means that a key concept in palliative care, quality of life, can never be achieved. To discuss symptom management, the regulator is not precisely defined. In systems theory, the regulator can also be a person within a group or family [39,41]. The decisive factor is that the regulator is part of the system and sets the goal on additional information from the system’s environment. When a regulator is assumed, it is important to note that there is no hierarchy in the system [10] but that individual structures can be compensated for by others (feedback [41]; limbic system and prefrontal cortex [63]).

According to Hell (2005), cortical centers, such as the prefrontal cortex (PFC), are no longer able to regulate the limbic system in patients with depression [41]. This finding is reflected in the psychiatric literature and even in genetics [46]. In the regulatory model of depression, the limbic system responds more strongly to medication, and the prefrontal cortex responds more strongly to psychotherapy [41]. From a cybernetic perspective, symptoms are failure of the control loop and feedback regulation. Therefore, the question for psychiatry is as follows: What resources could enable the control loop to function normally again?

#### 4.1.2. Regarding (B) (In)curability/Symptom Control

According to cybernetics (see Table 4), symptoms can only be reduced to the optimum level. In palliative psychiatry, optimization is often no longer possible from the perspective of chronic schizophrenia and treatment-resistant depression. This resulted in three patient groups: residual, treatment-resistant/treatment-refractory, and revolving-door patients.

*Residual:* Trachsel [3] does not explicitly mention residual depressive symptoms (therapy-refractory is not residual; of. Benkert and Hippius [21]) is in line with earlier academic thinking [64]. Bleuler [65], Kraepelin [66], and Leonhardt [67] assumed that depression always ended “well” [64].

From a cybernetic-psychiatric perspective, depression and schizophrenia are treated differently. The psychiatric literature from the last 30 years takes a different view of depression (Table 2). The figures show that in general psychiatry, not all patients can be treated until they are completely cured. One-third of patients achieve full remission, another one-third cope well, and the last third relapse [7,8]. This weighting does not (yet) exist for palliative psychiatry, where only patients who cannot be cured are the subject of discussion.

Trachsel et al. listed only residual symptoms in patients with schizophrenia (chronic schizophrenia). This is also the case in ICD-10 (F20.5) [3]. From a cybernetic-psychiatric perspective, neither the ICD 10 nor the ICD 11 recognizes residual depressive symptoms. According to Huber (2005), so-called “asthenic residues” can exist in depression symptoms [14]. These manifest as slight fatigue, vital malaise, poor concentration, reduced performance, increased impressionability, and depressive reactivity. Patients complain of a loss of drive, decisiveness, vitality, initiative, and stamina.

Schizophrenia also differs from concepts in the old school of psychiatry. According to Kraepelin and Wernicke-Kleist-Leonhard, schizophrenia always ends in residue [64]. Kurt Schneider and Huber [13] contradicted this [14] and showed that residual symptoms do not occur in patients with schizophrenia. In Huber’s study of the illness [14], 502 patients with schizophrenia were examined. After an average duration, 22.1% of patients achieved full remission, 43.2% had uncharacteristic residual symptoms, and 34.7% had characteristic residual symptoms. Patients with a “pure defect” also reported “somatic” symptoms such as body sensation disorders (coenesthesia), physical exhaustion, vegetative disorders, sleep disorders, and hypersensitivity to noise and weather conditions.

Both schizophrenia and depression may or may not result in residual symptoms. In patients with schizophrenia, a course without characteristic residual symptoms is possible if prodromal symptoms are considered symptoms and treated. These sometimes occur 30 years before the onset of primary symptoms, such as hallucinations.

*Resistance to therapy:* Trachsel and coauthors [3] cite treatment resistance only in cases of depression but not in cases of schizophrenia [21]. From a cybernetic psychiatric perspective, according to Benkert and Hippius, treatment resistance in depression can be identified [21] when two different antidepressants with different efficacy profiles are ineffective after 4–6 weeks of treatment at sufficient doses. According to Benkert and Hippius, resistance to antipsychotic therapy can also be assumed in patients with a confirmed diagnosis of schizophrenic disorder lasting at least 2 years if three different antipsychotics from two different classes at sufficient doses have been ineffective for at least 6 weeks and if psychotherapeutic treatment attempts have also been ineffective [21]. However, resistance to treatment does not mean that no further measures are possible, but that the usual treatment regimens are no longer effective.

The consequence of this definition of treatment resistance is that other methods of symptom minimization, such as ECT, augmentation, or light therapy, are pursued. However, this also raises the question of whether the treatment goals might need to be revised [6,68].

A Swiss research report cited the recovery concept [4]; 43.2% and 34.7% of patients had uncharacteristic and characteristic residual symptoms, respectively. According to observations, patients with mental illness and a “negative prognosis repeatedly succeed in leading a mentally stable and contented life and being professionally successful.” This should be viewed critically because, according to the abovementioned study by Huber [14], this is a reverse analogy to the lack of residual symptoms in depression [68].

At this point, it seems ethically questionable to no longer consider the prodromal stages/symptoms in schizophrenia in the ICD-11 (see criticism by Hölzel and Berger 2024 [69]) and, therefore, to be able to influence the course to the residual state only to a limited extent (because the illness has already lasted too long) and then make the schizophrenic residual state the subject of palliative psychiatry [70,71].

*Revolving-door patients:* The third group of patients (revolving-door patients) includes patients who have attempted suicide several times and who require repeated hospitalization [3,72,73,74]. This group also includes patients who frequently use psychiatric care [72].

#### 4.1.3. Re (C) Networking and Complexity

Here, palliative psychiatry is related to the areas of quality of life [48]: social relationships, family, and independence. In palliative medicine, Hodiamont et al. described that these are interconnected in psychiatry as important for patients’ quality of life: the social system and the “team system” [12,48]. From a cybernetic-psychiatric perspective, it is important to note that the combination of genetic-organic vulnerability, medical history (including social history), and acute stress factors leads to the development of schizophrenia and depression (cf. [27]). Recently, it has become increasingly apparent that pre-existing damage may also be genetic [46,53]. However, it is not assumed that depression or schizophrenia is caused by one or more genes but rather that it is the failure of counterregulation (a lack of adaptation of other biologically related genes) that leads to mental disorders [14,41]. According to Vester, networking is stable only if not all structures are simply networked with each other, but there are substructures [11,17]. Bender et al. cited the need to develop a systems theory for schizophrenia and referred to this as a “complex dynamic nonlinear system” [75] identified by Angermeyer et al.

#### 4.1.4. Re D: Autonomy

No comment is necessary from a cybernetic-psychiatric perspective. Please refer to Table 3 and Table 4, as well as previous publications on cybernetics [64].

#### 4.1.5. (E) Biopsychosocial

No comment is necessary from a cybernetic-psychiatric perspective. Please refer to Table 3 and Table 4 and the previous publication on cybernetics [64].

#### 4.1.6. (F) Quality of Life

Trachsel et al. [2016] reported that palliative psychiatry improves the quality of life of patients with severe mental disorders [3]. However, from a cybernetic-psychiatric perspective, the definition of quality of life must be critically questioned. According to Angermeyer, there exists a clear difference between some ideas of psychiatrists and patients [48]. While patients consider maintaining their lifestyle, psychiatrists are more oriented towards the absence of symptoms. From a cybernetic point of view, this can be seen in the various behaviors listed in Part III (see Figure 3).

A 2014 Swiss report cited the “recovery concept” in this context. This involves achieving satisfaction and well-being, a positive approach to one’s own illness experience, and full social integration (and recovery) [4] therapy. However, given the refractoriness, this is probably a difficult goal to achieve. The report also states that “patients should be able to lead a fulfilling and satisfying life despite persistent psychological symptoms by overcoming the influence of the illness” [4]. An important component of this is “empowerment”: through self-help and self-determination (autonomy), affected patients learn to deal with the illness in a positive way. Closely linked to the concept of autonomy and quality of life is the concept of dignity [6,9].

### 4.2. Overview of the Three Areas of Palliative Care, Psychiatry, and Palliative Psychiatry About Cybernetic Concepts

Cybernetics and its concepts point to errors in medicine as a whole. In earlier works, the consequences of assuming a networked cybernetic system have been noted [10]. This should now be supplemented by the question of which of these psychiatry, especially palliative psychiatry, already follows (see Table 5).

As shown in Table 5, psychiatry, as an older discipline than palliative care, has already contributed research and considerations to all cybernetic concepts. This applies in particular to specific studies on schizophrenia and depression. To date, palliative psychiatry has based its considerations on palliative care more than on psychiatry. Thus far, research in palliative psychiatry has made statements on symptoms, curability, networking/complexity, autonomy/biopsychosocial aspects, and quality of life, but these statements are incomplete in some respects (physical symptoms/symptom control, curability). This has consequences for palliative psychiatry from a cybernetic perspective. Neglecting physical symptoms also impacts other key areas, such as quality of life.

In addition, the following terms are missing in comparison to the statements discussed thus far (Table 5): cause-and-effect model, stability/identity, time (decay/temporalization, preformation/anticipation, rhythm), nonlinearity, fuzziness, and terms from the control loop model (i.e., control loop, dualism, self-regulation, autopoiesis, controller, resources, feedback control, and anticipation). It should also be noted that although the “recovery concept” is not explicitly mentioned, it is indirectly assumed to be a parameter for achieving structure, stability, and identity.

If we compare palliative psychiatry with palliative care, the main difference between the two is that in palliative care, most patients are those with tumors. In this context, the tumor is a qualitatively different disorder from the underlying disorder. However, palliative care also deals with nontumorous conditions (e.g., lung, heart, and neuromuscular diseases). Palliative psychiatry, as an aggravating course of an underlying disorder, is similar to care for nontumorous patients.

When comparing palliative psychiatry with general psychiatry, the following points emerge:

- In contrast to general and specialized psychiatry, palliative psychiatry is the younger discipline for the two diagnostic groups of schizophrenia and depression. It lacks knowledge of psychiatric teaching based on the ICD-10/11 and DSM-V diagnostic systems and attempts to take a theoretically descriptive approach to diagnosis. This leads to problems because the ICD-11 and clinical psychiatric reality do not correspond. For example, febrile catatonia, as described in the case study by Trachsel et al. (2022; in Westermair et al. [72]) and Elgudin [74], can also be a contraindication for the antipsychotic treatment of a specific mental disorder that no longer appears in the ICD-11: cycloid psychosis.
- To date, palliative psychiatry has been based on palliative care. As a result, only the terms biopsychosocial, quality of life, symptom control, and autonomy have been examined. Studies on terms such as nonlinearity, structure, and stability are lacking.
- With regard to work in palliative psychiatry related to schizophrenia, there are shortcomings outside the realm of cybernetic concepts. However, Irwin and coauthors have already lamented these shortcomings [76]: the stigmatization/disregard of patients [77], a lack of service providers who are familiar with this vulnerable group [78], and longer hospital stays and more medical orders for life-sustaining measures [79]. Levitt et al. call for better training for practitioners “to alleviate the anxieties of mental health care providers.” [80]. It must be respected that even ‘wrong’ decisions made by patients that do not correspond to the moral concepts of the nursing staff must be accepted [51]. As a result, patients with schizophrenia have a significantly lower life expectancy than patients with the same physical illnesses who are not diagnosed with schizophrenia. They differ by 10–20 years [81] or 30 years [82].

In summary, this means that palliative psychiatry is forced to acknowledge shortcomings in treating schizophrenia that have been known to general psychiatry since at least 2014. These shortcomings existed even before the advent of cybernetics.

In contrast to general psychiatry, there are no answers to cybernetics that relate to the malfunctioning of the control loop. Models for the genesis of schizophrenia and depression from a cybernetic perspective were developed as early as 1970.

### 4.3. Consequences

The following conclusions can be drawn from the discussion of this topic. The literature on palliative medicine/palliative psychiatry to date lacks work on cybernetic topics, especially in relation to schizophrenia (as well as depression), to answer the following questions:

- What does the disruption of the control loop/regulator mean for the treatment and therapy of schizophrenia and depression in palliative psychiatry?
- What does identity mean for treating patients with schizophrenia whose identity is disturbed?
- If stability in cybernetics also means decay, what does this mean for patients with schizophrenia, whose perceptions of the external and internal worlds are decaying [58]? Is it an overreaction of a natural process?
- If the biopsychosocial concept implies that behavior is not automatically wrong, what does this mean for nursing staff? [77]

Based on Vester [17], Brinkers et al. compiled seven errors in medicine and their cybernetic alternatives [83]. We present a supplement to what is already being done in general and palliative psychiatry (Table 6) and their alternatives.

Table 6 presents seven terms related to previous errors in psychiatric medicine. The left-hand column of the table lists errors that are frequently made in medicine—not always but repeatedly. A separate column (to the right of the errors) lists the attitude towards these errors. Cybernetics recommendations are to the right of this.

The errors are evaluated in detail below. What does palliative psychiatry do? Palliative psychiatry is based on terminology from palliative care. However, as we have established, this is not sufficient from a cybernetic point of view.

- General psychiatry clearly has more to say about a wide variety of cybernetic concepts than palliative medicine and psychiatry. Palliative psychiatry also draws on the basic knowledge of general psychiatry and implements it in part toward palliative psychiatry.
- In this case, palliative psychiatry has adopted the previous errors of psychiatry, namely, the failure to consider physical symptoms, as discussed in detail here.
- Simultaneously, palliative psychiatry introduces two new mistakes:
  - Irreversible focus formation (repeated administration of antipsychotics [84]) despite massive adverse effects [64].
  - Palliative psychiatry sometimes dispenses with interdisciplinarity [86]. However, in another study, no consulting psychiatrist had the additional designation of “palliative medicine” [85]. The lack of interdisciplinarity has already been reported elsewhere [81,87]. The authors also report that psychiatrists are generally not accustomed to acknowledging clinical futility and advocating for interdisciplinary collaboration.

The errors are now presented in a differentiated manner.

*Error No. 1: New subjective balance relative to the patient’s abilities*. There is no turning back in palliative medicine. The literature refers to the patient’s medical history. Therefore, one can only attempt to restore a new equilibrium based on the patient’s resources at present. This raises the following questions: Where can certain resources be strengthened with little effort? What can the patient still do? Thus far, psychiatry has provided few answers to these questions.

Psychiatry addresses various forms of residual symptoms. As seen in the table, error no. 1 and its alternative (no. 1) are not issues in psychiatry, as there is no restitutio ad integrum in CP.

*Error No. 2: Data reduction through pattern recognition was achieved.* Data accumulation occurs when several doctors contribute their expertise to a medical problem. In concrete terms, this means that tumor conferences often do not work in an interdisciplinary manner because each discipline contributes to the case only from its own perspective. Therefore, tumor conferences are multidisciplinary. Multidisciplinarity is only data accumulation. However, patterns should be recognized in the patient’s individual medical history.

In psychiatry, Ciompi describes the importance of structures as places of transformation and self-regulation [16] and equates structure with “system” (see also error 5). No work on this topic can be found in palliative psychiatry.

*Error 3: Alternative treatment options*. In addition to pharmacotherapy, psychiatry involves light therapy, sleep deprivation, and psychotherapy. However, in palliative psychiatry, a team may be too quick to determine what to do next. However, the important question is always whether there is an alternative to the current approach. Solutions for which no alternative exists are always suboptimal [64,84].

*Error No. 4: Additional impact assessment.* Impact to psychiatry assessment is important in several areas. Mistake no. 3 (lack of other treatment options) often leads only to the apparent solution [64,84]. However, when looking for a solution, the consequences that the action may have for the patient should also be considered. Husebo and Klaschik defined impact assessment as a measure of QoL [18].

Impact assessment has been practiced in psychiatry for decades and applies to the following three areas:

- Adverse effects of medication in general:
  ○ Teeth in antipsychotics (formerly neuroleptics) [88];
  ○ Obesity associated with the use of psychotropic drugs [89];
  ○ Withdrawal and rebound phenomena associated with antidepressants [90].
- Compliance: The Munich PIP study revealed that in the first year after hospitalization, approximately half as many patients were readmitted if they and their relatives had participated in psychoeducational groups [91].
- Suicidal tendencies are increased by medications such as selective serotonin reuptake inhibitors (SSRIs) [92].

Elgudin reported that impact assessment in palliative psychiatry is limited if one responds only to psychopathology but continues to administer antipsychotics that repeatedly produce the same psychopathology [84].

*Error No. 5: Treating a system, such as the musculoskeletal system.* When using psychotropic drugs, the focus is often only on symptoms without placing them in an overall context. An example of this is secondary, i.e., vitalized abnormal grief in patients with back pain. However, the more important question is where the pain comes from. Is it a consequence of the psyche or vice versa? This is the practical consequence of mistake no. 2 (lack of pattern recognition). In psychiatry, systems theory has been considered for years [16,39,41,57].

Palliative psychiatry has focused mainly on anorexia nervosa [73]. Articles on schizophrenia do not go beyond the terms used in palliative care. Palliative psychiatry has not yet addressed depression.

*Error No. 6: Creating an optimally insensitive system to disturbances (increasing reactivity).* In psychiatry, circadian rhythms continue to exist for treating depression, and patients may also experience depressive moods [14]. However, in contrast to untreated depression, it is important that patients are able to come out of their mood on their own with the help of therapy. Therefore, it is important to administer only enough antidepressants to alleviate physical and psychological symptoms.

Individual symptoms cannot be reduced to an absolute minimum. This would be possible, but at the expense of the rest of the body [40]. The more important question is as follows: What is the optimal level that a system (e.g., the cardiovascular system) can achieve at the present time? It is also important not to eliminate disruptive factors. In cybernetic systems, a disruptive factor is counterregulated.

Psychiatry describes several conditions in which the patient’s improvement comes to a standstill: maximization is possible. One is stuck in a rut. One reason for this may be that psychological symptoms are treated consistently, whereas physical symptoms are omitted [9]; therefore, patients continue to return [7,8,72]. These are therapy-resistant patients or patients with residual conditions (i.e., revolving-door patients) [72].

One possible approach would be to prevent the onset of residual conditions through relapse prevention and depot administration in patients with schizophrenia.

To date, palliative psychiatry has considered how to deal with patients. In the case of Elgudin [64,84], the focus is more on minimizing than optimizing previous therapy.

*Error No. 7: Interdisciplinarity: skills beyond one’s own profession instead of multiprofessionalism.* Following from mistake no. 2, what is needed is not a genius team with shared knowledge. This requires interdisciplinary work. However, a book on interdisciplinary palliative care published just three years ago presents the following definition: “interdisciplinarity” refers to skills beyond one’s own profession [93].

This means that only when doctors from different disciplines (e.g., all with additional training in palliative care and pain medicine) are working in these fields are they working in an interdisciplinary manner. This type of interdisciplinarity reveals patterns. For palliative psychiatry, this also means that psychiatrists and nurses can only progress with additional training [85]. Regardless of whether patients work exclusively with psychiatrists or nonpsychiatrists, they should have an understanding with palliative care specialists. The point is that psychiatry thus creates a common (interdisciplinary) basis. If we were to use a purely psychiatric vocabulary again without further explanation, it would not be useful for interdisciplinary work. This would be multidisciplinarity.

Etgen [2020] reported that palliative medicine in psychiatry is Unknown [13,85]. The lack of interdisciplinarity has already been lamented by Rosenbaum et al. [87] (Baruth et al. [81]). Rosenbaum et al. [87] noted that psychiatrists are generally not accustomed to recognizing clinical futility and advocated for interdisciplinary collaboration.

Thus far, no approach to interdisciplinarity has been developed in palliative psychiatry.

Moreover, mistake no. 7 (the lack of interdisciplinarity) in psychiatry without a fundamental structure is sought after by some palliative psychiatry authors [83]. However, interdisciplinary work is also a problem in palliative psychiatry, for example, in the case of pain. According to Bair et al. (2003, 2004) [94,95], pain is more common in patients with depression [94], just as pain increases depression [95].

The only problem here is that palliative psychiatry has thus far focused only on psychological symptoms. Quality of life requires that all symptoms reported by a patient be treated. This also includes somatic symptoms (e.g., pain) (see [6]). In this respect, the failure to address somatic symptoms in patients with schizophrenia and depression means that improving quality of life in palliative psychiatry is possible to a limited extent. According to the 2014 Swiss report, there is a lack of interprofessional/interdisciplinary cooperation [4]. Particularly because of somatic symptoms, internists should be integrated into the team, and psychiatric nurses should be better trained in somatic care.

Considering all the errors together leads to the following conclusions: Errors 2–6 have already been addressed in the psychiatric literature. However, in palliative psychiatry, there are no specific studies on errors 2 and 5. Errors 3 and 4 occur in palliative psychiatry. Error 6 is different: there are suggestions for an optimum recovery concept. However, at the same time, individual patients are offered the minimum [84].

In general, the focus of palliative psychiatry on palliative care rather than general psychiatry leads to an accumulation of errors.

All seven errors in the list are based on the desire to do something. Medicine always faces the difficulty of terminating treatment due to a lack of benefit, and psychiatry is no exception. This was the reason for establishing futility palliative care. The apparent opposite of the errors in Table 6 is the concept [87] only. If patients no longer benefit from a therapy or experience adverse side effects, the therapy should be discontinued. However, these seven errors indicate that this goal is difficult.

## 5. Conclusions

In this study, the use of cybernetic terms in general psychiatry (with a special focus on schizophrenia and depression) was compared with that in palliative medicine. In the Section 4, a comparison was drawn with research on palliative psychiatry about cybernetic terms.

General psychiatry, the older of the two psychiatric disciplines, has produced work on all of the cybernetic topics examined in this study.

As palliative psychiatry is the youngest of the three fields, it has, like palliative care, adopted only terms such as biopsychosocial, symptom control, quality of life, and autonomy from cybernetics. This has several consequences:

1. Palliative psychiatry has thus far focused excessively on the DSMV, ICD-10, and ICD-11 diagnostic systems. It has adopted the flaws of these systems, including the gap between them and everyday psychiatric reality. This is evident in the descriptions of the terms symptom/symptom treatment, quality of life, and incurability. Similar to general psychiatry, it does not consider somatic symptoms.
2. Moreover, palliative psychiatry introduces two new errors:
  - It is prone to irreversible specialization.
  - It partially dispenses with interdisciplinarity.
3. Specifically, the use of palliative psychiatry in palliative care for patients with schizophrenia remains outside the scope of cybernetic concepts. Instead, it is now “forced” to examine problems that have been known for more than 10 years as problems in the care of patients with schizophrenia, such as stigmatization, disregard for patients, or a lack of service providers who are familiar with this vulnerable group.

The use of cybernetic concepts in the psychiatric literature is, formally, better suited to gaining access to palliative psychiatry. At present, palliative psychiatry does not go beyond what palliative medicine has already achieved. However, palliative psychiatry should reflect on the work of general psychiatry and develop concepts such as complexity, nonlinearity, stability, identity, decay, and rhythm in the palliative care of patients with schizophrenia and depression.

The new “speciality” of palliative psychiatry increasingly focuses on patients who do not progress in therapy. Cybernetics may help clarify the requirements for approaching patients while including a culture of error management. An interdisciplinary approach is essential for achieving this goal. Palliative psychiatry should adhere more closely to the knowledge developed in the literature on general psychiatry.

## Figures and Tables

**Table 1:** Number of hits in PubMed: Cybernetics/cybernetic principles in psychiatry, schizophrenia and depression (search conducted on 18 February 2026).

Term	PubMed Number of Hits		
	Psychiatry	Schizophrenia	Depression
Regulator	111.780	14.335	50.920
Dualism	196	14	35
Autopoiesis	6	3	1
Feedback/counter-regulation	330	25	157
Biopsychosocial	298	31	203
QoL	36.583	6.183	69.607
Symptom/symptom treatment	207.208	50.191	207.807
Autonomy	5.061	477	2.322
Cause and effect	103.706	28.844	124.245
Nonlinearity	14.045	2.122	12.450
Complexity/ networking	48.641/ 51.891	10.801/ 9.605	32.963/ 27.914
Structure	79.635	16.132	56.816
Identity	10.464	987	6.074
Stability	14.675	3.994	12.657
Time terms	49.079	9.433	59.331
Noncybernetic terms:			
Palliative care	4.685	152	4.942
Genetics	101.484	32.038	52.428
Summe	839.767	185.367	720.872

**Table 2:** Number of hits in PubPsych: Cybernetics/cybernetic principles in psychiatry, schizophrenia, and depression (search conducted on 18 February 2026).

Term	Number of Hits in PubPsych Database		
	Psychiatry	Schizophrenia	Depression
Regulator	612	180	208
Dualism	169	22	22
Autopoiesis	10	2	---
Feedback/counter-regulation	53	5	17
Biopsychosocial	1645	191	947
QoL	19,459	4281	19,546
Symptom/symptom treatment	13,266	3638	9299
Autonomy	2869	402	1082
Cause and effect	2294	511	1104
Nonlinearity	75	9	28
Complexity/networking	4522/18,984	775/4061	1143/7179
Structure	2018	3967	7718
Identity	9310	874	2995
Stability	5209	1203	2515
Time terms	3300	660	1426
Noncybernetic terms:			
Palliative care	1699	60	1175
Genetics	42,644	14,497	10,767
Total	146,307	35,338	67,171

**Table 3:** List of important statements from the literature review for cybernetic terms, differentiated according to cybernetics, palliative care, and general psychiatry.

Cybernetic Terms	Cybernetics [11]	Palliative Care [11]	General Psychiatry
**Cybernetics, palliative medicine, psychiatry and literature**			
**Biopsychosocial**	Cybernetic systems are inherently dualistic [11].	The biopsychosocial model is commonly accepted in medicine [13].	Genetics does not fully explain the clinical picture of mental disorders [14,15,16]. The biopsychosocial model must be supplemented with the diathesis-stress model [14]. The essence of a disease is [15] a nonlinear interaction of system components [14]. According to Ciompi [16], there exists a dependency on the outside world. Total shielding from all sensory stimuli quickly leads to a profound decline in consciousness, reaching psychotic levels or resulting in death.
**Quality of life (QoL)**	One way to improve quality of life is to eliminate errors in previous procedures [17].	The impact of the assessment is on quality of life [18].	QoL is difficult to define precisely [19]. According to Bullinger [20], QoL is a health-related aspect of personal well-being in the physical, mental, social, psychological, and functional spheres. This means that important factors include the individual’s perception and processing of the disease, in addition to conditions in their living and working environments.
**Symptoms**	Symptoms begin at the tipping point (when resources are exhausted) [11].	Symptoms arise from loss of counterregulation.	In particular, physical symptoms also occur in relapse [7,8].
**Symptom control**	Disturbing factors are not eliminated. The system is made insensitive to the disturbing factor. The goal of therapy is to achieve a new balance based on QoL and satisfaction, as well as the creation of new resources for compensation [11].	In therapy, the narrow sense is a reduction of symptoms as long as the patient can tolerate it, not to the status quo ante, which cannot be determined [11].	There are three aspects to the treatment of symptoms: prevention, rapid remission of acute symptoms, and recurrence consideration of adverse effects [21].
			Symptoms can still be treated in the residual phase [14].
**Autonomy**	Autonomy is not self-sufficient. Autonomy means energetically open or procedurally closed. Instead of an information monopoly, communication should be based on agreed rules. No action or attitude is a priori better than another. Only adherence to the rules is correct [11,22].	Patient autonomy refers to the desire to continue to be able to carry out the activities of daily life and to have control over one’s life, in accordance with one’s existing belief systems, personality, and values [23].	Psychiatric autonomy is a core concept [24]. There are three concepts: (1) An interpersonal relationship based on mutual recognition. (2) An ethic that respectfully and interactively focuses on the life history and values of the person being cared for. (3) Autonomy as an individual ability embedded in a relational context.
**Cause and effect**	Owing to the nonlinearity from the outset, there is no fixed relationship between cause and effect [11].	Palliative medicine refers to noncurative treatment [25,26].	A pure one-cause, one-effect principle is not possible because of the often multiple factors involved [27].
**Cybernetics and literature on psychiatry outside palliative care** From this point on, palliative medicine rarely uses these terms. However, the literature on psychiatry in general and on mental disorders does.			
**Nonlinearity**	The original state cannot be inferred from the current state [11].	No publication	Even if all biopsychosocial factors are known, predicting a patient’s decision is impossible [28]. The interaction of individual factors in comorbidities is nonlinear [29]. Different initial states can lead to the same [16] or psychopathological expression community [14].
**Complexity of systems/** **Interconnection**	From a cybernetic perspective, systems are complex when their parts are connected by mutual, constantly changing relationships (networking). Complexity does not require hierarchy [11]. Only the development of substructures stabilizes the system [11].	Complexity as the assessment of patients (physical, psycho-spiritual, socio-cultural), social system, and team [12].	Cumulative dysregulation at the neuronal-endocrine, immunological, metabolic, and cardiovascular levels leads to increased vulnerability to mental disorders [30,31]. The interconnection of cerebral structures has long been a topic of discussion in psychiatry [32]. A system can be defined as a “set” of elements that are interrelated, whereby the state of each element is determined by that of other elements [16].
**Structure**	Owing to the vagueness, the function does not require as much data as possible, but only a data pattern/structure [11].	No publication	Structure is the (…biological, social, psychological) regularity that has developed during its functioning and behaves according to a system of rules [33]. Structures can usually be understood as a composition of substructures, which are part of higher-level structures in a network of influencing factors [33]. The structure and system are virtually identical [16]. According to Rudolf et al., the concept of system emphasizes the dynamic processes of homeostatic balance of states in the sense of control loops, while the concept of structure emphasizes the aspect of genesis [33]. The structure is not rigid and unchangeable but shows lifelong development processes. According to Ciompi, structure is a system of transformations [16].
**Identity**	Derived, among other things, from stability [11].	No publication	The concept of “I” can be defined by four characteristics: the activity of the I, the unity of the I, spatial and temporal continuity, and the independence of the I (I am only myself—never someone else) [34].
**Stability**	Cybernetics is not a sequence of individual operations but a process [11].	No publication	Stability means that fluctuations in the psyche can be balanced out by the individual, or that preventing psychological fluctuations only lasts for a short time. Stabilizing this state means relapse [21] and providing patient education. Therapy-resistant schizophrenia can be stable [14]. Life is a mixture of stability and instability [11,16,35].
**Time factor**	Owing to autonomous processes, there may be a time difference compared to the outside world [11].	No publication	The altered subjective experience of time is found in many mental disorders [36]. According to Ciompi [16], any significant deviation from the usual “mental tempo” leads to extremes, such as excessive fixation of attention on certain details that would otherwise be hardly noticed, combined with stretching, shortening, or other alteration of the experience of time.
**Decay**	Decay and reconstruction are necessary systems in the constant synchronization between the goals and results of the processes [11].	No publication	Patients with decay of ego structures occur in schizophrenia [14].
**Rhythm/Oscillation/Fluctuation**	The psyche is subject to daily and seasonal fluctuations [11].	No publication	The sleep–wake cycle is a particularly sensitive structure in mental disorders [16,37].
**Anticipation**	The existence of preformed systems enables the system to respond to future demands [11].	No publication	According to Bilz, the human ability to anticipate events (in terms of time [38]) is a cause of anxiety.

**Table 5:** Cybernetic concepts in palliative care, psychiatry (including schizophrenia and depression), and palliative psychiatry.

Cybernetic Terms	Equivalent in …		
	Palliative Care	General for Psychiatry and Patients with Schizophrenia and Depression	Palliative Psychiatry
**Biopsychosocial**	+	+	+
**QoL**	+	+	+
**Symptoms/** **Symptom control**	+	+	+
**Autonomy**	+	+	+
**Cause and effect**	+	+	---
**Nonlinearity**	(+)	+	---
**Complexity/interconnection**	(+)	+	+
**Structure**	---	+	no publication
**Stability**	---	+	no publication
**Identity**	---	+	no publication
**Time factor**	---	+	---
**Decay/temporalization, preformation/anticipation**	---	+	---
**Rhythm**	---	+	---

**Table 6:** Typical errors in medicine and how psychiatry and palliative psychiatry have acted to date.

Mistake No.	Error Content	What Should Be Done Instead?	What is Being Done	
			Already Being Performed in General Psychiatry	In Palliative Psychiatry
**1**	Goal: Restitutio ad integrum	New subjective balance relative to the patient’s abilities	Residual symptoms instead of restitutio ad integrum	Courses may be incurable (i.e., no restitutio ad integrum).
**2**	Data accumulation	Data reduction through pattern recognition (PR)	Structure [16] = Coexistence of variance and invariance	No work on pattern recognition.
**3**	Irreversible focal point formation	Alternative treatment options	Antidepressants and/or antipsychotics: light, sleep deprivation, psychotherapy	This error occurs in palliative psychiatry. [64,84]
**4**	Solving the problem alone (e.g., removing the painful area)	Additional assessment of consequences	Adverse effects of medication, compliance, suicidal tendencies	Incorrect treatment due to unrecognized mental disorder = palliative [64,84]?
**5**	Pain treatment as a disruptive factor (known as Dawos’ rule)	Treatment of a system, such as the musculoskeletal system	Structural model = flexibility [16] = autoregulation	Work on anorexia nervosa as a system [73]. No equivalent for schizophrenia or depression to date.
**6**	Maximization as a goal	Create a disorder-insensitive system (increase reactivity); optimal therapy	Through medication to prevent recurrence and depot administration for schizophrenia; psychoeducation	What is the optimal? See Swiss report: Recovery concept [4].
**7**	Specialization	Interdisciplinarity: skills beyond one’s own profession instead of multiprofessionalism	Interdisciplinarity is not present [85]	Errors: Currently, palliative medicine in psychiatry is provided by psychiatrists [86], of. [87]. There are also considerations regarding interdisciplinarity/interprofessionality [4].

## Data Availability

No new data were created or analyzed in this study.
